# Cloud-Native Workload Orchestration at the Edge: A Deployment Review and Future Directions

**DOI:** 10.3390/s23042215

**Published:** 2023-02-16

**Authors:** Rafael Vaño, Ignacio Lacalle, Piotr Sowiński, Raúl S-Julián, Carlos E. Palau

**Affiliations:** 1 Communications Department, Universitat Politècnica de València, 46022 Valencia, Spain; 2 Systems Research Institute, Polish Academy of Sciences, ul. Newelska 6, 01-447 Warsaw, Poland; 3 Warsaw University of Technology, pl. Politechniki 1, 00-661 Warsaw, Poland

**Keywords:** edge computing, cloud computing, edge-to-cloud computing continuum, edge-native, cloud-native, container, Kubernetes, microVM, Unikernel, WebAssembly

## Abstract

Cloud-native computing principles such as virtualization and orchestration are key to transferring to the promising paradigm of edge computing. Challenges of containerization, operative models and scarce availability of established tools make a thorough review indispensable. Therefore, the authors have described the practical methods and tools found in the literature as well as in current community-led development projects, and have thoroughly exposed the future directions of the field. Container virtualization and its orchestration through Kubernetes have dominated the cloud computing domain, while major efforts have been recently recorded focused on the adaptation of these technologies to the edge. Such initiatives have addressed either the reduction of container engines and the development of specific tailored operating systems or the development of smaller K8s distributions and edge-focused adaptations (such as KubeEdge). Finally, new workload virtualization approaches, such as WebAssembly modules together with the joint orchestration of these heterogeneous workloads, seem to be the topics to pay attention to in the short to medium term.

## 1. Introduction

The leading technological companies have been focused for the last ten to fifteen years on developing their systems and services in “the cloud”, where computing capabilities and infrastructure are offered remotely on demand. It involves different concepts depending on the requested service: Infrastructure as a Service (IaaS), Platform as a Service (PaaS), Software as a Service (SaaS) and Functions as a Service (FaaS). A characteristic of this paradigm is that the supporting hardware (which is usually located in datacenters) is highly homogenous. Usually, all machines that are part of a cloud deployment have uniform configurations, for instance, the same ad hoc operating systems that might have been customized by vendors to achieve a better global performance bearing in mind the type of service provided. High network bandwidth and reliability are often guaranteed in cloud computing as well. However, there is a recent observable trend to move the computation to more local environments, favoring the so-called edge computing. Here, devices located at the edge tier of the computing continuum are charged with more processing duties, reducing the work to be delivered by the cloud. In this environment, the underlying computing hardware is entirely diverse; heterogeneous in CPU architectures and constrained in resources, not fully controlled by system owners (less customizable and less powerful) and deployed in the field, where network connection is often non-reliable [1]. Although much work and effort is being put towards realizing this new paradigm, it is still undeniable that cloud computing has a grade of maturity not achieved by the edge [2]. Looking widely at the currently existing gaps, there are two that stand out: orchestration and Continuous Integration/Continuous Deployment (CI/CD). Cloud computing has achieved a high level of automation thanks to well-explored techniques ranging from virtualization, containerization, container orchestration, microservices or DevOps, to CI/CD pipelines, which allow cloud deployments to be very dynamic and efficient. The relevance of these features has made the community refer to them as cloud-native traits. Such techniques, despite having many different implementations, can be considered vendor-agnostic de facto standards, starting from the most relevant single tool: Kubernetes (K8s). Thus, the gap is evident when comparing both paradigms, as there are no equivalent edge-native techniques yet, or those are in an early development stage. The goal of such techniques should be to devise vendor-agnostic standards that would allow efficient CI/CD and orchestration of services in the same way that has been created for the cloud, and as much as possible, recycling or adapting those to the edge, taking advantage of their long-term production stage. The goal of this paper is to perform a review from a deployment point of view of the progress exerted by the scientific community to realize such cloud (now, edge) native techniques for the edge computing paradigm.

The review has been performed following a deployment-first orientation together with a literature analysis methodology. First, the specific scope of the paper was clearly identified. Afterwards, a series of searches in usual article databases considering the target knowledge domain (such as IEEE, Elsevier, Springer, Google Scholar), among which results were selected with those that included similar reviews or analyses. In parallel, a thorough analysis of alternative sources was conducted. Among those, authors priorities open repositories (e.g., GitHub tools and collections of reference sources), deliverables and whitepapers distributed by research projects and relevant, well-known clusters or standardization entities (such as Cloud Native Computing Foundation or Alliance of Internet of Things Initiative). Once those efforts were performed, the analyzed pieces were categorized by the type of virtualization addressed, whether they presented (or not) commercial approaches, focused on orchestration, focused on the deployment of workloads, and those that were focused on future trends and promises rather than on current cases.

This paper is organized as follows. In Section 2, a succinct analysis of the context and the compelling need for such a review is carried out. Section 3 reviews the first and foremost cloud-native technique to be applied to the edge: service virtualization and deployment in constrained devices. Section 4 provides an overview of the holistic commercial solutions that have been specifically addressed to the edge from a cloud-native perspective. After that, Section 5 lists the deployment options of the most common type of service workloads (containers) within the edge paradigm, while Section 6 analyzes the orchestration of those containers using frameworks such as K8s. In Section 7, the authors describe the most recent trends that are paving the way for the future of edge-native deployments: moving beyond containers only. Finally, Section 8 embodies the reflections and conclusions obtained by the authors after the conducted review.

## 2. Background

Edge-native as a concept was first introduced by Satyanarayanan et al. in 2019 [3]. After that, it has been used in multiple fora by companies such as IBM, Cisco, and others, settling as the most popular term to refer to the application of cloud-native design principles on edge computing devices. These cornerstone principles are microservices-centricity (achieving modular distributed system deployment), containerization, dynamic management, orchestration and scheduling, and DevOps [4].

There is a growing interest in research on transferring technologies and tools to the edge to comply with such principles, applying agility, adaptability, affordability, and accessibility traits to edge scenarios. This interest is mostly manifested by the launching of so-called projects, particularly those promoted by renowned open-source initiatives like Linux Foundation (LF Edge line [5]) or Eclipse Foundation (Edge Native Working Group [6]). Especially relevant is here the Cloud Native Computing Foundation (CNCF), the vendor-neutral hub that has standardized and fostered cloud-native principles and technologies like Kubernetes, *etcd*, CoreDNS, gRPC, and many others. CNCF has launched a series of incubated and sandbox projects under their umbrella specifically pursuing edge-native goals [7]. In addition, ad hoc funding tenders have been scheduled by public entities such as the European Commission (EC) [8], such as the development of meta operating systems (meta-OS) to be installed in edge computing nodes [9], or distributed smart orchestration of various workloads across the edge and cloud computing fabric using Artificial Intelligence (AI) [10]. The former aims at realizing the so-called IoT-edge-cloud computing continuum, which roots its success on applying cloud-native techniques along the entire computational spectrum. Following the open-source spirit of Eclipse and CNCF, the EC has launched an ambitious initiative: EUCloudEdgeIoT.eu, that aims to bring together all European research actions seeking to devise such computing continuums with a common stack of open-source technologies [11].

It is precisely under the IoT-edge-cloud computing continuum working action where this article was born. Authors have participated in two EC-funded actions investigating aspects related to edge computing. First, the ASSIST-IoT research and innovation action [12] pursues to develop a reference architecture for next-generation Internet of Things (IoT) leveraging cloud-native orchestration (i.e., using K8s distributions) to manage IoT workloads and provide specific advanced features. Second, the aerOS action [13] intends to build a generic meta-OS (to be run by many heterogeneous nodes) that lays infrastructure management on secure, trusted, and automated AI mechanisms. During the former, the authors identified a series of CNCF-promoted tools and lightweight K8s versions, which might be applicable across the continuum; however, there has not been enough grounding or proof of success to consolidate a single reference yet. Through the latter, authors are investigating how to simultaneously harmonize edge and cloud clusters using different virtualization and workload management techniques.

The authors found several recent surveys [2,14] describing the actual status of edge computing. Those range from general analyses of architectures and potential use cases to narrowly focused research on specific aspects of the edge, such as security [15], networking [16] or mobile edge computing (MEC) [17,18]—mainly addressed by telecom operators more concerned to 5G innovations. Found surveys and reviews often compare edge to cloud, but only a few delve into the deployment and orchestration options [19], which is the scope of this work. In fact, a common reality was faced; first during both formerly described EC-funded actions and then after the review of existing relevant works: namely, the lack of a serious review of the available options for bringing containerization, orchestration and workloads management to the edge in a way that is adapted to the requirements of heterogeneous, resource-constrained scenarios. It was noticed that even though there exist some theoretical investigations [4], as well as specific descriptions of how some elements (e.g., containers) should be adapted to the edge [20], a practical, systematic consolidation of the existing deployable technologies with a summative evaluation was still missing. The previous, in turn, prevents scientists and engineers from tackling the practical problem of realizing cloud-inspired virtualization and orchestration of the edge in a straightforward manner. Addressing such a problem is, indeed, of special importance as it lies right at the core of the current challenges and issues of the edge computing field. Among the most compelling challenges, there is reliability assurance in terms of security in data exchange, better DevOps and CI/CD and network availability for microservice operations [21]. Besides, finding an operating model seems to also be one of the most prominent barriers. According to works, that could be alleviated by applying a combination of the already-successful microservices and cloud-native principles in the edge [22]. As a note, for the foreseeable future, overweight imposed by usual container management frameworks might also be a challenge. Thus, this article was initiated.

The main motivation behind this work is to allow those specialists to understand the current options and issues in a holistic way, as well as to provide critical insight into the short- and medium-term trends. It is worth mentioning that, being a deployment-oriented review, it has gone beyond pure scientific literature analysis (that would most likely transpire outdated results) and has broadened its scope to other sources such as open-source development projects or successful use cases. Indeed, agile, dynamic opensource software initiatives are now the forerunners to the advances in this field. Thus, scientific reviews must include this perspective in the analysis so that it is up-to-date and provide useful insights for the research community. Consequently, this strategy seems to be appropriate for this research field, as the outcomes are usually more likely to be published in public code repositories, blogs or websites (primarily from reference institutions such as the CNCF and enterprises such as Docker Inc., Palo Alto, CA, USA) rather than in formalized scientific publications.

## 3. Virtualization: From Cloud Computing to Constrained Environments

One of the main keystones behind the success of cloud computing is the capacity to virtualize the available resources and the ability to manage workloads (services/applications) in an efficient way over virtualized hardware [23]. Virtual Machines (VMs) were the first attempt, enabling the creation of smaller instances of machines over the same physical infrastructure. This paradigm dominated the cloud computing arena for several years until new approaches appeared aiming to solve several shortcomings of VMs. First, the overhead created by the need to install multiple OSs to support different VMs was hindering the potential to devote those resources to actual workloads. In addition, the workloads became very diverse and heterogeneous (video streaming, sensor data processing, AI…), requiring dynamicity in the installation and management, something that VM hypervisors were not able to provide [24]. Furthermore, the utilization of hypervisors required system owners and developers to be aware of the needed resources and the infrastructure to be selected to deploy their services or applications. Several initiatives appeared with the aim of overcoming those issues, having major success in the cloud computing field. In this subsection, those initiatives are described from the viewpoint of edge computing.

To solve the latter problem (required knowledge of underlying hardware and resources), a paradigm arose called *serverless*. *Serverless* architectures are widely adopted by public cloud providers, offering their customers the possibility of running their applications without having to take care of: (1) the infrastructure where it will be really deployed and (2) for how long to reserve it. This paradigm has been especially successful in the public cloud as it builds upon the assumption that the available computing resources are well identified (e.g., as in datacenters) and that the infrastructure is easily manageable and accessible in a uniform way by a single operator (cloud provider). However, these principles are exactly orthogonal to the reality of edge computing, where a software architect cannot omit the insights regarding the infrastructure when an application’s deployment must take into account the underlying heterogeneity of resources. Notwithstanding, some initiatives for applying *serverless* mechanisms to the edge have arisen. Especially relevant are those that rely on the temporal dynamicity of services deployment in order to downscale or upscale on demand the resources devoted to services, optimizing resource consumption (which is of paramount importance on the edge). Examples following this approach are projects such as OpenFaaS [25] and Knative [26], which take advantage of the Kubernetes technology (see Section 6). Knative (which is an incubating project of the CNCF) also provides a complete event-driven engine based on CloudEvents, a specification to standardize event data descriptions, which opens a wide range of possibilities in K8s-based architectures on the edge [27]. With the inclusion of this event-driven engine, the deployed services can trigger different events to activate some functionalities or workloads without modifying the source code.

To solve the former problem (overheads caused by installing one OS per each virtual machine), a paradigm arose called container virtualization. Container virtualization implies the use of containers to isolate applications and their dependencies into smaller units that contain the service to be deployed. The ruling principle of container virtualization is that no hypervisor (plus OS per machine) is needed; in contrast, the host’s single OS is virtualized, allowing containers to be deployed isolated, but still sharing the same kernel [28]. This makes containers much more lightweight and scalable. Because of all these advantages, containers have become the de facto standard for running virtualized deployments in the cloud (cloud-native) and also are the natural evolution of the legacy VM-based deployments [29]. Several container technologies exist (e.g., LXC [30]—LinuX Containers—was one of the first developed container runtimes), but since Docker was released in 2013, this technology has been the standard solution for this virtualization technique. Docker has been successfully tested and adopted in production-grade deployments around the industry’s public and private clouds for different purposes. The objective of this review work is not to explain the functioning of the containers along with the technology that is behind them (there are strong works in the literature aiming at this purpose, such as [24,29] or [31]), but to review from a deployment perspective how these mechanisms are used in the field of edge computing. Although the hardware limitations of edge devices can make it impossible to run the Docker Engine on them (which is the most used container engine and has remained the de facto standard for the container virtualization), authors have found that containers are widely used in edge scenarios with a variety of deployment options, as described in Section 5.

It should be noted that the area of systems based on cloud-native computing principles is evolving rapidly to meet the shifting demands of the industry. Current challenges and open issues include unified orchestration for heterogeneous systems, federated management domains, leveraging artificial intelligence for automatic resource management and data interoperability [32,33]. Interestingly, the surveys also acknowledge that one of the main challenges is applying the existing cloud-native principles to the entire edge-to-cloud continuum.

## 4. Current Commercial Approaches from Public Cloud Providers

Most public cloud providers have addressed edge computing as an extension of their own cloud infrastructure scope. Consequently, the majority of available solutions from those companies consist of their standard commercial cloud products, but assuming customers’ premises as the central cloud locations. In addition, those providers have also devised and created their own hardware devices for the edge adapted to run their new (cloud) edge solutions. This way, cloud providers made sure that the selected equipment would be plug-and-play, prepared to run their products, keeping their advantages and service level with zero configuration needed from the final user. However, apart from creating huge vendor lock-in environments, most of these frameworks are not actually edge-native solutions responding to the relevant requirements; they only shift workloads outside the cloud infrastructure but without breaking a strong dependency on the cloud or adapting architectural paradigms to the edge. This section analyzes those solutions that are provided by the large public cloud providers.

Amazon Web Services (AWS) offers a solution named AWS IoT Greengrass for deploying processing capabilities over nodes at the edge tier of the architecture, especially over IoT devices that gather data from various attached sensors [34]. This solution includes *serverless*-based deployments in the devices using AWS Lambda, the *serverless* approach of AWS. It transforms models that were previously created in the cloud into Lambda functions, that are then shifted to the edge (where container virtualization and AI inference equipment exist) to be run. To avoid network connectivity loss problems, Greengrass creates a virtual twin of the device that ensures compliance with the desired state in the cloud, as long as there is a reliable connection; otherwise, it re-synchronizes whenever it is re-established and calibrates to reassure the desired state again. In addition, inter-device communication is allowed inside a local network without depending on the cloud. As for device management, AWS IoT Greengrass is highly configurable from a remote location, allowing the addition and removal of (even customized) hardware and software modules. Another solution by AWS for the edge is AWS Snowball Edge, a device type from the AWS Snowball family that is designed to work at the customer’s premises [35]. Amazon offers three versions of such a device that aim to enhance different demanded capabilities: storage-optimized for data transfer (80TB of usable storage capacity), storage-optimized with EC2 compute functionality (AWS product focused on proving computing capabilities on demand) and compute-optimized (with 104 vCPUs and 416 GB of memory). Similar to Greengrass modules, Snowball edge devices can be managed locally and through the cloud. Furthermore, they can run powerful workloads to move all locally stored data to the AWS S3 storage service in the cloud and control the deployments running on the Greengrass devices. Finally, these devices can be classified as the top layer of the edge tier of the computing continuum because they provide great computing capabilities, approaching a small cloud datacenter.

Microsoft Azure provides a complete stack for edge computing under its Azure IoT Edge framework, including remote equipment management, edge-level virtualization, and remote workload allocation and control [36]. This technology is delivered with an open-source MIT license via the GitHub account of Azure, allowing developers to deploy and integrate Azure’s edge stack in their own infrastructure. However, interoperability is not complete, as the installation depends on the Azure cloud stack. Both Azure and AWS have delivered several series of their own certified devices targeting the lowest and medium tier of the edge computing range (some of them are based on popular embedded boards, such as Raspberry Pi, NVIDIA Jetson and Intel boards). This certification consists of the validation by the Azure cloud engineers that a device “can connect with Azure IoT Hub and securely provision through the Device Provisioning Service (DPS)” [37]. Similar to Amazon, Microsoft offers a line of powerful equipment to bring the Azure services to the customer’s premises and remote locations (near the sources of data), lowering the amount of data to be uploaded to the cloud by the Azure Stack Edge. This equipment is divided into two lines of products: Edge Pro Series, a line focused on powerful products to be located both in a local datacenter (Pro and Pro 2), and transportable equipment that can contain an uninterruptable power supply (Pro R); Edge Mini Series, a constrained portable device with a battery [38].

Google Cloud integrates edge computing solutions under its Google Distributed Cloud solution. Google devised a complete edge-to-cloud continuum infrastructure by offering services that can be deployed on all of their own-defined tiers of the edge-to-cloud architecture layers (Google’s network edge, telecommunications operators’ edge, customers’ datacenters, and customer’ remote edge), including the telecommunication service provider network layer and the possibility of virtualizing telcos’ 5G network elements [39].

A common aspect of all the mentioned proposals by prominent cloud providers is the lack of interoperability and the high degree of vendor lock-in. Whereas the mechanisms and technologies that will be introduced in further sections (K8s distributions, *KubeEdge*, and other orchestrators) are open and oriented to comply with certain baselines of cloud-native principles, the commercial systems presented here create closer ecosystems tied to interdependencies, specific configurations, particular ways of structuring and deploying applications, etc.

## 5. Container Virtualization: Deploying Container Workloads on the Edge

In 2015, the Open Container Initiative (OCI) was founded by the container technology leaders (Docker, CoreOS, …) in order to specify a standard for container virtualization. This action resulted in the creation of three specifications: the Runtime Specification (*runtime-spec*, the specification for running containers), the Image Specification (*image-spec*, for packaging containers in images) and the Distribution Specification (*distribution-spec*, for sharing and using container images) [40]. Two years later, when the Docker engine was established as the technological reference for containers, Docker open-sourced the code of its container platform, divided into modules (including its high-level container runtime—*containerd* [41] and its low-level container runtime—*runC* [42]), with the purpose of bringing the capability of building fully customized container-based systems to system builders. This innovation received the name the Moby Project [43]. It allowed engineers to focus on the development of lighter container runtimes through the reduction of their internal components and the removal of unnecessary modules in order to increase their performance or even to design specific operating systems incorporating such runtimes, which appear to be interesting trends for both cloud and edge. Subsequent subsections dig deeper into these two approaches and the initiatives that have been identified for edge computing scenarios.

### 5.1. Container Engines

**Figure 1:**
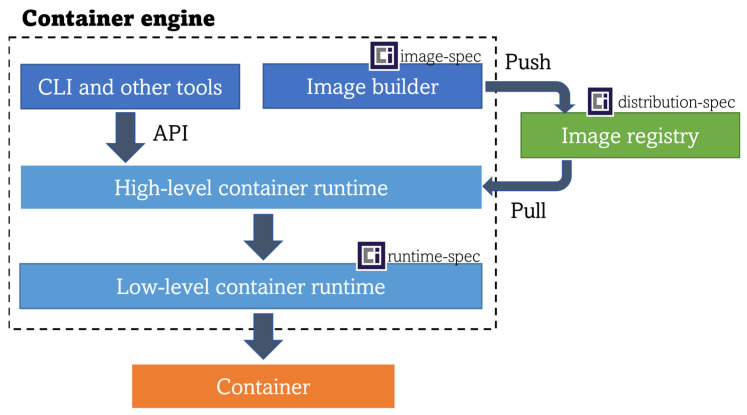
Container workflow block architecture.

Container engines (e.g., Docker, Podman) are comprised of tools for building and running container images. To achieve the latter, a high-level container runtime (also referred to as the container manager, this block controls the transport and management of images) interacts with a low-level container runtime (the block that receives the unpackaged container image from the high-level runtime and finally runs the container inside the system interacting with the host’s OS kernel), as shown in Figure 1. As mentioned in the previous paragraph, Docker Engine’s runtime (de-facto standard) materializes in *containerd* and *runC*. However, there are other runtime implementations that have been developed with edge computing constraints in mind.

*crun* is a low-level container runtime fully compliant with the OCI runtime specification that is written in C, the same as LXC and the Linux kernel, unlike *runC*, which is written in Go. Here, *crun* improves upon *runC*, as C can bring better performance than Go. Furthermore, the *crun* binary is vastly smaller than the *runC* binary (300 KB versus 15 MB). As a matter of fact, in some studies *crun* has proved to perform better in a test consisting of running sequentially 100 containers. In the test, *crun* achieved the goal in 1.69 s, while *runC* took 3.34 s [44].

Rust is a programming language that released its 1.0 version in 2020; since then it has been established as a strong alternative to C and C++ due to remarkable improvements in memory safety. Rust also betters Go in the implementation of system calls, which has pushed its usage to develop *youki*, an OCI-compliant low-level container runtime that shares with *crun* the reduction of binary size, making both suitable for environments with constrained resources. In a similar test performed for *crun*, *youki* showed a better performance compared to *runC*, while *crun* remained as the fastest one [45].

Focusing on the high-level runtimes, *CRI-O* appears to be one of the more interesting solutions. It is a lightweight container runtime specifically designed for Kubernetes [46]. Its main advantage is the reduction of resource consumption in comparison with Docker’s *containerd*, Moby or *rkt* (also analyzed), and its compatibility with any OCI-compliant low-level runtime, an interesting feature that allows this runtime to run workloads not only based on containers, a promising trend that is covered in Section 7 of this article. However, *CRI-O* has not been developed for use in low-resource embedded devices, requiring the target device to be able to run Kubernetes. Therefore, there remains much research to do in the field of high-level runtimes specifically designed for edge-tier devices.

### 5.2. Purpose-Built Operating Systems

In the same way that container runtimes are being reduced to target a wider range of systems, some specific OSs have appeared that take advantage of reduced and portable runtimes with the main goal of bringing container virtualization to increasingly constrained, as well as embedded devices.

EVE-OS is an operating system originally developed by ZEDEDA and then donated under an open-source license to the Linux Foundation that has included it inside its edge research projects stack (LF-Edge) [47]. The main purpose for the development of this OS is to provide edge computing devices with a universal, vendor-agnostic and standardized architecture OS, following the same strategy that Google used in the smartphone market when it delivered Android. EVE-OS has adopted well-known open-source projects such as Xen Project, LinuxKit and Alpine Linux for its development. Currently, the remote management of a fleet of devices running EVE-OS following a Zero Trust security model is possible using the Adam controller, the reference implementation of an LF-Edge API-compliant Controller. Furthermore, EVE-OS provides support for running containerized workloads using *containerd* and for running Kubernetes distributions on top of it. EVE’s contributors have recently announced an architecture proposal for integrating EVE with Kubernetes at a control plane level [48]. The OS is designed for edge devices with reasonable computing capabilities (minimum of 512 MB of RAM memory) and has been prepared to be deployed in a wide range of CPU architectures. Moreover, EVE-OS has enough functionalities to be deployed on bare metal and supports a wide range of workloads that can be combined: Docker containers, Kubernetes clusters, and virtual machines.

BalenaOS is a host operating system with the purpose of running Docker containers on embedded devices and has been made to survive harsh networking conditions and unexpected shutdowns [49]. The OS is based on Yocto Linux, which provides a small memory footprint and the possibility of easily porting it to more powerful devices across a variety of CPU architectures [50]. Its purpose is to replicate cloud operating systems’ applications deployment in edge computing devices through the use of containers (cloud-native to edge-native). For this purpose, Balena has developed its own container engine (*balenaEngine* [51]) based on Moby project technology. The engine was adapted through: (1) the removal of the “heavy” Docker features oriented to the cloud (Swarm, plugin support, overlay networking drivers…) not suiting edge nodes, and also (2) through the addition of particular features specifically intended for less-powerful computing devices (support for the most common CPU architectures on single-board computers, improvement of container image pulling to prevent network failures…). At this moment, the OS is supported by up to 20 device types.

The Balena company offers as a service Balena Cloud, an automated platform to manage infrastructure running BalenaOS and the workloads deployed in such devices, which has been optimized for the edge. Last but not least, Balena has delivered this management software in an open-source way for advanced users or infrastructure managers that want to host this platform on their own premises without depending on Balena. By using this platform, developers are able to deploy application containers, push updates, check status, and view logs of the fleet of devices that have been previously registered.

According to the Pantacor company, Docker was built without considering embedded devices, as it requires a high level of resource availability. In order to relax those requirements, they developed Pantavisor, a minimal low-level container runtime written in C that shares commonalities with *crun* (getting closer to the Linux kernel) [52]. Pantavisor has the purpose of shifting systems into a set of portable microservices (materialized in Linux containers) instead of the traditional scheme of firmware plus applications. According to Pantacor, Pantavisor “is meant to be a single-binary init system that boots directly from the kernel and becomes the first process to run, which then brings up the rest of the system as a set of well-defined micro-containers” [53]. This container runtime is compatible with ARM, MIPS, RISC-V, x86, and PowerPC CPU architectures, has a running footprint of just around 350 KB and requires a minimum of only 64 MB of RAM.

In contrast with the traditional container-based architectures, Pantavisor does not need a container runtime running on top of a complete OS. This is more suitable, then, for embedded devices as they might not use all features of a host OS. Thus, by containerizing the host OS layer, Pantavisor transforms it into a container, embodying the traits of updatability, scalability, etc. In this architecture, Pantavisor acts as the minimal container runtime manager of the system. Same as Balena, Pantacor provides a framework (*PantacorHub*) to manage the devices running Pantavisor and the workloads deployed on them. As an interesting note, it is offered either as an open-source tool for self-hosting or as a paid service hosted by the company itself.

## 6. Container Orchestration Tools for the Edge

Although containers can be managed using their own interfaces, some tools have appeared on top of container virtualization technologies to better manage and orchestrate the deployment, allowing scheduling, scaling and control. Kubernetes (K8s) has become the standard for container and microservices orchestration in the cloud, gaining advantage over its competitors in recent years, such as Docker Swarm or Apache Mesos [54]. Consequently, as K8s is a highly customizable and open technology, most cloud providers have delivered their own K8s distributions that are fully compliant with the standard in order to optimize the integration of K8s with their systems. As reviewed in the previous section, bringing containers to edge computing deployments is possible, and it is in fact being implemented. Therefore, to transfer cloud-native traits provided by a *standardized* container orchestration tool (K8s) to the edge, an equivalent technology—adapted to edge computing—must be found. This is a relevant point, as Kubernetes itself does not seem to be the proper solution. Although its deployment is feasible in some computing nodes along the computing continuum [55,56] (those that can carry powerful workloads), it does not suit the capacities of more resource-constrained equipment such as far-edge nodes or leaf devices. In some works (e.g., [57,58]), this problem is introduced along with some suggested alternatives. The scope of this section is precisely to analyze potential candidates to become the K8s alternative for container orchestration in edge-computing deployments.

**Figure 2:**
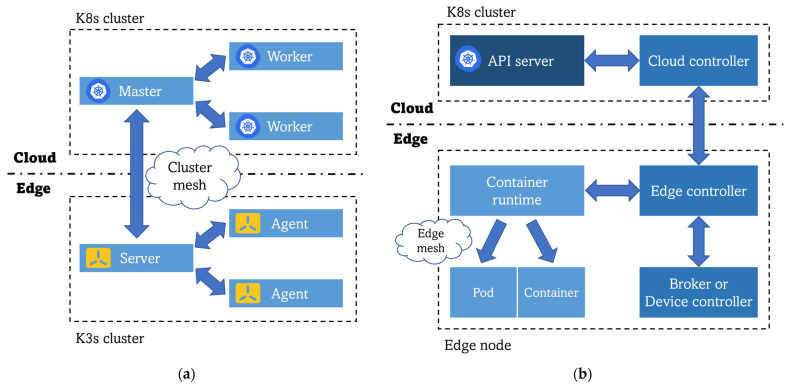
Comparison of architectures for K8s deployment at the edge: (**a**) K8s multi-cluster architecture using lightweight K8s distributions for the edge clusters; (**b**) K8s adapted to the edge computing requirements general architecture.

The solutions that have been discovered in state-of-the-art investigations are divided into two main groups. The first group focuses on replicating Kubernetes in edge environments by reducing the memory footprint of this orchestration platform to broaden the range of equipment in which K8s can be fully functional. Here, different lightweight versions of Kubernetes can co-live with a full K8s deployment in the cloud premises, creating a sort of multi-cluster environment so that communication and orchestration in the computing continuum is achieved. This approach is illustrated in Figure 2a. On the other hand, Figure 2b depicts the second trend of creating new frameworks specifically adapted to the edge that follow Kubernetes principles but are not direct simplifications or reductions of that technology. These solutions adapt K8s to the specific characteristics of the edge (unstable network connectivity, difficulty of managing heterogeneous and low-resource equipment), maintaining all its benefits achieved in the cloud and not just limiting themselves to creating another lightweight K8s distribution without reaching a real edge-to-cloud synergy.

In the first group, *K3s* stands out. *K3s* is a lightweight, fully compliant Kubernetes distribution developed by Rancher. It was created for running in constrained devices, bearing a much lower memory footprint than other available K8s distributions [59]. *K3s* modifies the K8s paradigm of master and worker nodes, converting them into server and agent nodes. On another note, it offers three possible architectures: (1) a single server with an embedded SQLite database and (2) high-availability servers using an embedded or (3) an external database (SQL-based or *etcd*). This distribution is also optimized for several CPU architectures, such as ARM32, ARM64, and ARMv7. Henceforth, it becomes preferrable for edge environments (which often use embedded systems as nodes, like Raspberry Pis or NVIDIA Jetson boards). *K3s*’s minimum requirements are 256 MB of RAM for an agent node, and 512 MB for a server node with some workloads running in the agent node. Rancher also delivered an OS for edge nodes optimized for running *K3s*, maintaining only minimal resources of the underlying OS: *k3OS* [60]. Its low memory footprint and the ability to run on devices having diverse CPU architectures converts *K3s* into the most recommended K8s distribution for building clusters on the edge following the first approach, as mentioned above. It is also supported by the Cloud Native Computing Foundation. As an alternative lightweight K8s distribution, Canonical released *MicroK8s*, which claims to have a minimal memory usage of around 540 MB, but its recommended memory allocation is 4 GB, which is considerably more than *K3s* [61]. *MicroK8s* is clearly less mature and proven than *K3s*, and according to the authors’ experience, it is not yet ready to be considered a robust option for pure edge-native deployments [62]. Nevertheless, *MicroK8s* has the advantage of being an easily customizable distribution (via *add-ons* installed by simply executing its *install* command). The available *add-ons* include some of the most widely used K8s modules, such as CoreDNS, Helm or Istio, and the possibility of achieving K8s High Availability features in a straightforward way. These features make *MicroK8s* one of the most interesting K8s distributions for development and testing in slightly more powerful edge devices (especially those with more than 1 GB of available RAM). These two edge-focused distributions have been compared between them and to a complete K8s distribution installed using Kubespray in [63], giving as a result, a clear performance improvement against a classic distribution without showing significant performance differences between *K3s* and *MicroK8s*. Therefore, there is not yet a de facto standard equivalent for K8s on the edge, but *K3s* holds the advantage of more popularity, better robustness, and a lower memory footprint. It is worth mentioning that these lightweight distributions are specifically designed to deploy K8s-like clusters on the edge tier of the architecture, but they could perfectly work in cloud clusters since these distributions are completely functional and certified by the CNCF. However, a classic Kubernetes installation with K8s clusters is preferred for the cloud premises. As a matter of fact, in order to realize the cloud-to-edge continuum with cloud principles, both topological locations in the architecture should comprise K8s(-like) clusters, materializing the so-called *multi-cluster* scenario. Multi-cluster techniques are commonly used for enabling an edge-to-cloud synergy between clusters deployed along the edge-to-cloud continuum, but this paradigm comes with a number of drawbacks. Due to the fact of being designed for cloud environments, the inner mechanisms of multi-cluster tools (e.g., communication, networking, high-availability, services discovery…) are not currently adapted to the particularities of edge computing. Nonetheless, this is an active research field, and there are some projects and initiatives investigating possible solutions. An illustrative example can be found in the EU H2020 research project ASSIST-IoT, where a K8s-based multi-cluster architecture is devised, in which clusters (*K3s* is recommended for edge premises) are registered to a smart orchestrator. The orchestrator is in charge of connecting the clusters leveraging Cilium Multi-cluster technology [64], selecting the optimal placement of applications in the computing continuum based on AI policies, and managing their lifecycle [65].

**Figure 3:**
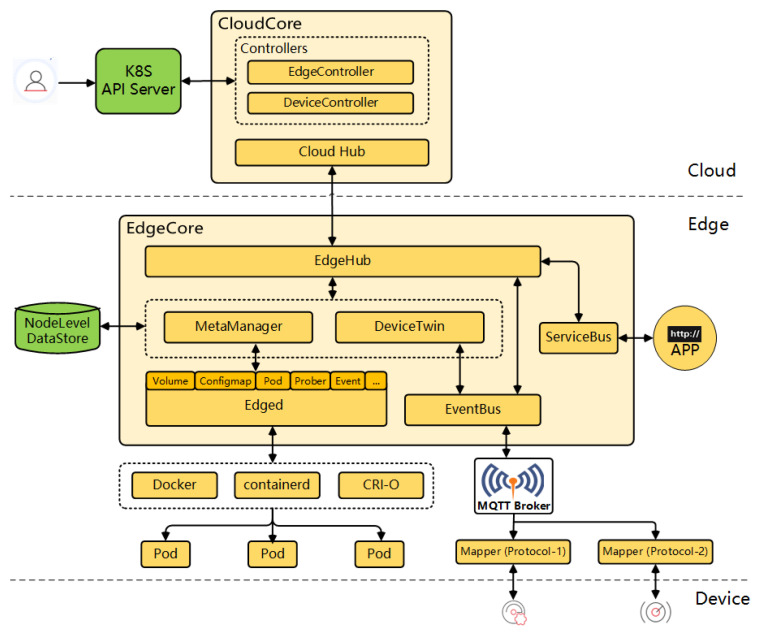
*KubeEdge* architecture [66].

In the second group, the design of the deployment differs ostensibly. Some of the devised new frameworks that adapt K8s principles to the edge are based on maintaining the control plane of the system in the cloud, and moving only the needed workloads and specific controllers to the edge, converting it into an autonomous node of the system regarding the application plane. So minimized, essential information from the control plane is cached on the edge or in intermediate nodes in order to be accessible in case of network connectivity issues. Among the analyzed solutions of this type, *KubeEdge* stands out. *KubeEdge* [66] is an open-source framework based on the Kubernetes architecture with the main purpose of bringing the full functionalities of Kubernetes to the edge. This ongoing deployment project is officially under the umbrella of the CNCF. The main underlying idea of this technology is to keep the entire control plane in the cloud, where the computing resources are more plentiful, and leave the workloads (running the containers) or the application plane to the edge in order to dedicate all the constrained computing resources of this tier for this purpose. Moreover, the task of controlling the communication with far edge devices without real computing capabilities (sensors, cameras, …) is assigned to the K8s edge nodes. This together translates into a low memory footprint of the *EdgeCore* installation (the edge part of *KubeEdge*) of only around 70 MB. The *KubeEdge* architecture is divided into three layers:

- Cloud: for *KubeEdge* to work properly, there is a need to have a running K8s distribution in the cloud that will interact with a *CloudCore* instance (the cloud part of *KubeEdge*) deployed on the same host. This cloud part of *KubeEdge* includes controllers to synchronize the status of all edge nodes and leaf devices connected to the nodes.
- Edge: the components deployed inside the *EdgeCore* handle communication between application containers, connected devices, and the cloud tier. The K8s pods (workloads that are orchestrated) are deployed in this layer, but its deployment is controlled by the cloud. The main difference as compared to a lightweight K8s distribution is that the edge part of *KubeEdge* is not a K8s node itself, disposing of the K8s API and its control plane. The inner components of *EdgeCore* are illustrated in Figure 3, taken from the official site of *KubeEdge*. In addition, the edge node in a *KubeEdge* deployment includes an MQTT broker to interact with device mappers (the available device protocol mapper types are Bluetooth, Modbus, and OPC-UA, but a Go library is provided to allow developers to create mappers for other protocols). The mappers are in charge of the interaction and control of the leaf devices, as well as of their lifecycle management.
- Devices: leaf devices with almost no computing capabilities. They interact with the edge layer through the mappers using various industrial protocols for data exchange.

**Figure 4:**
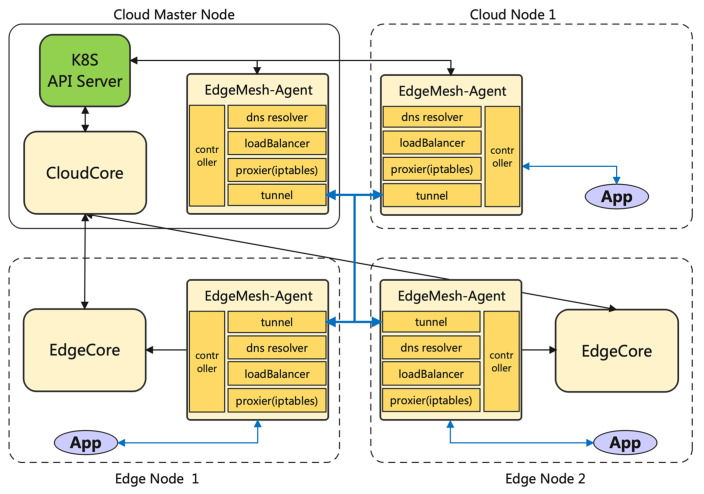
*KubeEdge*’s *EdgeMesh* architecture [67].

A remarkable feature of *KubeEdge* specifically intended for edge-native deployments is the capability of coping with poor network connection between the cloud and the edge. Synchronization is handled only under stable network conditions, thus achieving a smooth management of the environment. This is a key issue for edge-native applications that is not natively solved by K8s, as non-cloud aspects were dismissed in its original design. In addition, *KubeEdge* also provides the so-called service mesh capabilities. The service mesh provides service-to-service communication and discovery for all services deployed on any layer of the continuum as long as those services are controlled by the same cloud master node. The service mesh feature allows for transparent communication in complex network environments and establishes high-reliability scenarios foreseeing potential network issues through the distribution of network metadata across all the EdgeMesh Agents and the integration of lightweight DNS servers. Its architecture is illustrated in Figure 4 (extracted from *KubeEdge*’s official site). Initially, *KubeEdge*’s *EdgeMesh* was a part of the *EdgeCore* component (see Figure 3), but currently, it is delivered as a separate element that can be installed in all edge nodes in order to enable its deployed services to join to the service mesh [67].

*KubeEdge* has successfully gone through a series of scalability tests which demonstrated that the framework was capable of orchestrating one million pods deployed across 100K edge nodes, accomplishing the K8s Service Level Indicators (SLI) and Service Level Objectives (SLO). The solution managed the entire infrastructure as a true continuum represented by a single K8s cluster [68]. Similarly, an illustrative use case built using *KubeEdge* is the deployment of more than 100K monitoring devices across the Hong Kong–Zhuhai–Macao bridge, one of the longest bridges in the world. This work was presented during the *Kubernetes on Edge Day Europe 2021* by Huan Wei [69]. The edge tier of *KubeEdge* is deployed in every device located along the bridge, and all those devices are managed in a centralized manner by the cloud part deployed in a public cloud datacenter. Each monitoring device gathers data from 14 different sensors (CO_2_, PM2.5, temperature, humidity, …) through its specific mapper, and the data is processed locally using AI inference algorithms deployed on the edge nodes. Only selected data is finally uploaded to the cloud through a reliable 5G connection, but in case of network issues, following the offloading of the control plane strategy, a cache mechanism on the edge assures that no data is lost in the process. Taking advantage of the edge-to-cloud synergy achieved in the *KubeEdge* project, the same community of developers tried to use this technology to improve the execution of Artificial Intelligence workloads in the edge-to-cloud continuum. For this purpose, they have developed Sedna, a project focused on implementing collaborative training and inference capabilities across the edge-cloud continuum [70].

Although *KubeEdge* is the main reference of the second category of approaches, it is not the only such framework in existence. The CNCF accepted as sandbox members two more projects: *OpenYurt* [71] (the first open-source project carried out by the Chinese giant Alibaba) and *SuperEdge* [72] (whose realization is led by Tencent along with Intel, VMware, Huya, Cambricon, Captialonline, and Meituan). Both *OpenYurt* and *SuperEdge* are more focused on edge datacenters or relatively large edge devices due to their higher computational requirements as compared to *KubeEdge*. One of the most remarkable features of *OpenYurt* is the integration with the EdgeX Foundry for edge device management [73]. With regards to the required communication between the control plane (in the cloud) and edge nodes, these two projects share the implementation of secure tunnels that overcome the underlying connection obstacles caused by the interaction of several heterogeneous networks to which edge nodes may belong. According to Alibaba, the principal difference between *OpenYurt* and *KubeEdge* is that the former is less disruptive with the K8s architecture, as it only enhances K8s through the usage of plugins and operators, while the latter attempts to rewrite some components such as *kubelet* or *kube-proxy* [71]. Another interesting technology for bringing container orchestration to the edge is *Open Horizon* (OH); it was first developed by IBM and then donated to the Linux Foundation [74]. This technology shares with *KubeEdge* the concept of moving the workloads to the edge tier of the architecture but maintaining the control plane (workload and edge devices management) in the cloud or in a centralized environment. Furthermore, OH promises to manage up to 10,000 edge devices simultaneously from a single Management Hub instance. *Open Horizon* architecture is divided into two main components: (1) Management Hub: located at the centralized cloud in which a K8s distribution must be running. It oversees the control plane regarding the deployments and the edge nodes and devices. (2) Edge Agent: this component is divided into two subtypes depending on the workload type that will be run by the node. The Edge Device Agent is targeted for resource-constrained devices which are capable of running containerized workloads through a container runtime, while the Edge Cluster Agent is appropriate for equipment in which a K8s distribution can be installed. *Open Horizon* is also gaining popularity among the edge-native community, as the EVE-OS developers (introduced in Section 5—both are official projects of the Linux Foundation) are planning to support OH-based workloads (K8s pods are natively supported). *Baetyl* is another project which shares some key concepts with *KubeEdge* and *Open Horizon* since its architecture is split into: (1) the Cloud Management Suite and (2) the Edge Computing Framework [75]. However, *Baetyl* only supports edge nodes with a minimum of 1 GB of RAM that are capable of running a K8s distribution (*K3s* is recommended for resource-constrained environments), not being yet capable of orchestrating container workloads by itself. This tool is also included in stage 1 of Linux Foundation Edge, and thus it also seems to be promising, although, at the moment of this review, it is in a preliminary stage with a clear lack of documentation.

At the beginning of this section, it was explained that there are two major types of approaches for materializing container orchestration at the edge, both following the principles and implementation of the cloud-native reference in the cloud: Kubernetes. However, there also exist a few other frameworks that separate from the K8s *standard* but that also address such orchestration. A representative example of these alternatives is *ioFog*, developed by the Eclipse Foundation. This framework presents some similarities with Kubernetes, such as the use of the *iofogctl* command and the specification of microservices using YAML files. It also has commonalities with other edge-specific technologies mentioned in this section, such as having an architecture based on deploying a controller in the cloud in charge of the agents running in the edge devices, which in this case is named Edge Compute Network [76].

After reviewing all these technologies, Table 1 has been created to illustrate the differences among the most relevant container orchestration tools for the edge, paying attention to selected key aspects.

To conclude this section, the authors deem it interesting to refer to the *Akri* framework, which is also under the CNCF umbrella, as a Sandbox project. *Akri* focuses on the development of a Kubernetes Resource Interface that allows exposing a range of heterogeneous leaf devices located at the lowest tier of the continuum as resources in a K8s cluster, for instance, IP cameras or USB devices connected to the same machine that is a K8s node [77]. This is not a K8s lightweight distribution, nor does it aim at orchestrating containers at the edge level of the architecture. It is only a component that complements an edge instance by allowing the very end devices to be recognized from high-level management frameworks, providing a layer of abstraction for the devices in a similar way the CNI does for the network. This is the main difference between *Akri* and the technologies presented above (*KubeEdge*, *Open Horizon*, …), and the reason why it is not included in Table 1. However, it is relevant to be considered from the viewpoint of a deployment review of the cloud-native approach on the edge. Technically, leaf devices interact with the *Akri* Agent service running on the nearest K8s node of the cluster, and thus *Akri* extends the K8s functionalities but does not adapt it to edge-native scenarios in contrast to, e.g., *KubeEdge*. *Akri* relies on a set of device Discovery Handlers based on management and communication protocols for industrial devices such as ONVIF, *udev* and OPC UA, as well as the possibility of extending this set with custom handlers [78]. When a new device is discovered by the handlers, *Akri* creates a K8s service to monitor its state and provide high availability in case a node loses network connection or breaks down.

## 7. Future Directions: The Horizon beyond Containers-Only

**Figure 5:**
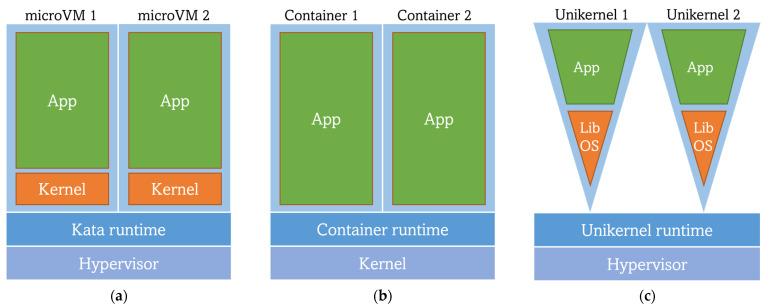
Comparison of the architectures of different virtualization techniques: (**a**) MicroVMs lightweight VM; (**b**) Containers; (**c**) Unikernels.

Section 3 has concluded that nowadays, cloud-native implementations revolve around orchestrating workloads in the form of containers, taking advantage of container virtualization techniques. Undeniably, this is the current de facto standard. However, containers are far from being established as the final and definitive approach in a technological field where everything is in continuous change. In fact, it is already well-known that containers are not perfect since they present some weaknesses (e.g., in security [79]) and have a reduced capacity for improvement over their current state. Regarding the utilization of containers in edge computing scenarios, although they are widely adopted at the moment (see Section 5 and Section 6), they still have a downside in their required technological stack and in the large working memory footprint and container image sizes that they have. Therefore, other virtualization techniques that could compete with containers have appeared, becoming current trends in the deployment research field. According to works that compare containers, VMs and Unikernels [80], new paradigms are arising (see Figure 5). However, this does not necessarily mean that these technologies will substitute containers in the short term, but they can simply be seen as a powerful complement for containers, and either one or the other may be used in specific use cases and, most likely, together.

In Section 3, a short review of the advantages of containers versus VMs is presented. While those arguments do hold true, the isolation capabilities of the latter (due to not sharing the same kernel among virtualized instances but using a hypervisor) are interesting from an edge computing perspective. Separating the instances at a lower level may allow for better security of user workloads, as, in contrast with containers, there would not be multi-tenant untrusted environments. Realizing the previous findings, a new approach for workload virtualization has arisen, keeping the isolation trait while skipping some inconveniences of the VMs. This is reached by reducing the size and requirements of VMs, achieving the so-called microVMs or lightweight VMs that intend to be even lighter and faster than containers. However, no clear results to date would demonstrate this reliably. Some works [81] have compared the performance of container (*runC*) and microVM (Kata Container) runtimes, showing better performance of the former. It was argued that this underperformance might be caused by the longer boot times of microVMs (3.7 s on average), as compared to containers (0.633 s on average). The microVM technology utilized for the comparison (Kata Containers) is an OpenStack tool whose main goal is to run lighter VMs instead of containers while remaining compliant with the OCI runtime and image specifications (see Section 5). By sticking to OCI, Kata allows Docker images previously built from Dockerfiles to be deployed as microVMs (Kata containers) [82]. This underperformance has been addressed through the development of a lighter container runtime oriented to running microVM-based workloads, but also OCI-compliant: *RunD*, especially focused on solving the bottleneck in *serverless* function deployments. *RunD* has been adopted by Alibaba as its *serverless* container runtime, serving more than 1 million functions and almost 4 billion invocations daily. Furthermore, *RunD* has been able to start 200 lightweight VMs in a second and to successfully deploy 2500 of them in a machine with 384 GB of memory [83].

Another approach to replace or complement containers with microVMs are Unikernels. According to [84], “Unikernels are single-purpose appliances that are compile-time specialized into standalone kernels and sealed against modification when deployed to a cloud platform”. The main idea beyond Unikernels is to use only the strictly necessary part of the user and kernel space of an operating system to obtain a customized OS that will be run by a type 1 hypervisor. Such customized OS is achieved through the usage of a library operating system (library OS or libOS), removing the need for a whole guest OS [85]. This is translated into a reduction of image size and their booting time, as well as their footprint and possible attack surface. However, this virtualization technology has many disadvantages, the most prominent being the lack of standardization as compared with containers, in addition to the need of a complete library operating system rebuild for every new application with any minimal changes, followed by the limitation of debugging and monitoring capabilities [86]. Unikernel applications are also language-specific (there are Unikernel development systems only for a few languages), being also a considerable limitation for developers. As an example, MirageOS is a library OS that builds Unikernels using the OCaml language together with libraries that provide networking, storage and concurrency support [87]. Nabla containers is an IBM research project focused on building a platform to handle Unikernel workloads (for instance, workloads built using MirageOS) through the usage of its own low-level container runtime *runnc* [88,89]. While *runnc* is OCI-compliant, Nabla’s image specification is not, thus, software packaged with container image specifications other than Nabla’s one is not supported by this technology. In addition, unfortunately, the Nabla project seems to be in a frozen state. Notwithstanding, newer approaches to Unikernels have emerged with the aim of making this paradigm a reasonable option with better integration in the cloud-native virtualization environment. *Unikraft* is a Linux-based “automated system for building Unikernels” under the umbrella of the Linux Foundation (inside its Xen Project) and partially funded by the EU H2020 project UNICORE [90]. While other similar initiatives exist, such as Lupine, *Unikraft* presents an improved performance in comparison to its peers and other virtualization technologies, such as Docker (regarding boot time, image size, and memory consumption). *Unikraft* leverages an OCI-compliant runtime (*runu*) along with *libvirt* (a toolkit for interacting with the hypervisor) for running Unikernels. The novelty is that *runu* natively supports the execution of workloads previously packaged following the OCI Image Specification (unlike Nabla), enabling Unikraft-built Unikernels’ interaction with containers and cloud-native platforms like K8s [91,92]. However, this promising Unikernel approach is still in a preliminary stage and is yet to cope with some challenges regarding, for instance: (1) the improvement of Unikernel applications packaging as OCI images and (2) the distribution of those images in OCI registries (e.g., the support of images for different CPU architectures). Furthermore, the source code of *runu* has not been delivered yet due to its instability and it being in a continuous improvement stage [93].

One step ahead of MicroVMs and Unikernels is the most recent and most promising trend in workload virtualization: WebAssembly (Wasm). Wasm is “*a binary instruction format for stack-based virtual machines*” developed as an open standard by the World Wide Web Consortium (W3C), that seeks to establish itself as a strong alternative to containers. Wasm allows software to be written in several high-level languages (C++, Go, Kotlin, …) to be compiled and executed with near-native performance in web applications that are designed to run in web browsers [94,95]. Still, some works have been conducted following the SPEC (Standard Performance Evaluation Corporation) benchmarks and have shown an underperformance of Wasm as compared with native code by a factor of 1.45 for Firefox and 1.55 for Chrome [96]. This technology was first designed to enhance the performance of demanding applications that perform poorly when written in JavaScript instead of languages specifically tailored for its working purpose. At a high level, the functioning of Wasm is simple: native code functions are compiled into Wasm modules that then are loaded and used by a web application (in JavaScript code) in a similar way to how ES6 modules are managed. Related to these pre-built modules, the work of [97] has researched the possibility of using them as a newer obfuscation technique for inserting malware inside web applications, making it harder to detect by malware analyzers. This drawback can be seen as an example of the new risks that groundbreaking technologies always bring with them.

**Figure 6:**
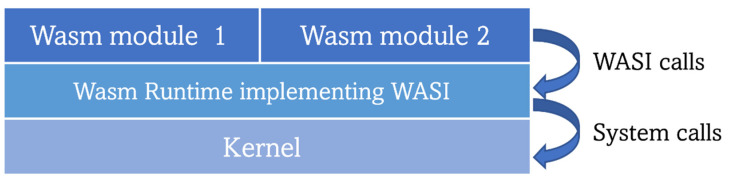
Architecture for running WebAssembly workloads on the server side.

In recent times, developers have attempted to move Wasm outside web browsers, or, in other words, from the (web) client side to the server side for a standalone mode of operation. Wasm binaries’ tiny size, low memory footprint, great isolation, fast booting (up to 100 times faster than containers), and response times make Wasm perfect for running workloads in edge and IoT devices. Adopting Wasm in edge environments (where resource-constrained nodes struggle to run container workloads) could lead to an increased number of simultaneously running services as compared with the current container throughput in the same environment [98,99]. Digging deeper into Wasm functioning, it is worth noting that when a Wasm binary is executed in the web browser, it uses the web APIs provided by the browser to interact with the required external components through appropriate system calls. However, when engineers started trying to run Wasm modules outside the web framework, an equivalent of such an API did not exist. It was required to establish such a “bridge” software to enable the interaction between a Wasm module and the host operating system calls. Thus, efforts were focused on delivering a standard set of APIs prepared to facilitate standalone Wasm module deployment. This was done while upholding two main Wasm principles: portability, which means being able to run the same code across different systems, and security. This research led to the creation of the WebAssembly System Interface (WASI), a “modular system interface for WebAssembly” with the main purpose of enabling the execution of Wasm on the server side through the creation and standardization of APIs. Realizing the above-mentioned security concerns, WASI was carefully designed to minimize the attack surface and prevent malicious Wasm modules from compromising the host system. Furthermore, WASI was designed to be independent of the used Wasm runtime (the component in charge of running Wasm modules or applications, it can be feasibly compared with the low-level container runtimes in terms of functionalities as both are the components that actually run the workloads) [100,101]. Wasm modules make WASI calls to a Wasm runtime that are then translated into the appropriate system calls to finally interact with the OS kernel [102], as depicted in Figure 6. Nowadays, WASI is still in the process of being standardized, promoted by a subgroup of the WebAssembly Community Group of W3C through a set of standardization proposals (HTTP, filesystem, machine learning, …). The proposals are grouped into 5 phases of development following the Phase Process of the aforementioned community group. Currently, the most mature proposals are in phase 2, which can be seen as another indicator of this technology’s novelty [103].

Naturally, WebAssembly runtimes are an essential element of the Wasm stack, since, at the end of the day, the execution of Wasm modules relies on them. In recent years, several Wasm runtimes have been developed that move the execution of Wasm workloads outside the browser. Interestingly, most of them have realized the potential in the edge-cloud continuum field, as they are specifically adapted to work in edge computing scenarios. These runtimes differ from each other in the supported WASI specifications and in the providence of programming SDK or implementation APIs to let developers load Wasm modules in the source code of their applications, among other aspects. An interesting, identified feature is that some runtimes (*Wasmer* [104], *Wasmtime* [105] and *WasmEdge* [106]) are natively supported in the low-level container runtime *crun* to achieve integration with containerized workloads, a trend that will be explained in detail later. For a better understanding of the differences among the existing runtimes, and with the final purpose of becoming a helpful resource for developers, Table 2 has been created, which compares carefully selected Wasm runtimes.

Apart from the industry, some works related to Wasm runtimes are being conducted in the research world. In [102] is presented *Wasmachine*, an OS that tries to move the WASI specification to its kernel written in Rust (the benefits of Rust compared to C have already been discussed in Section 5), so it can avoid the overload of the system calls introduced by the Wasm runtime in order to achieve a faster performance compared to native code directly executed on Linux. The results presented in this paper show an increase in the execution speed of 21%. On another note, *Twine* is a Wasm runtime resulting from the interaction between the industry and the H2020 research project VEDLIoT due to the fact that it is based on *WAMR* (see Table 2) but more focused on embedded and trusted environments [107].

**Figure 7:**
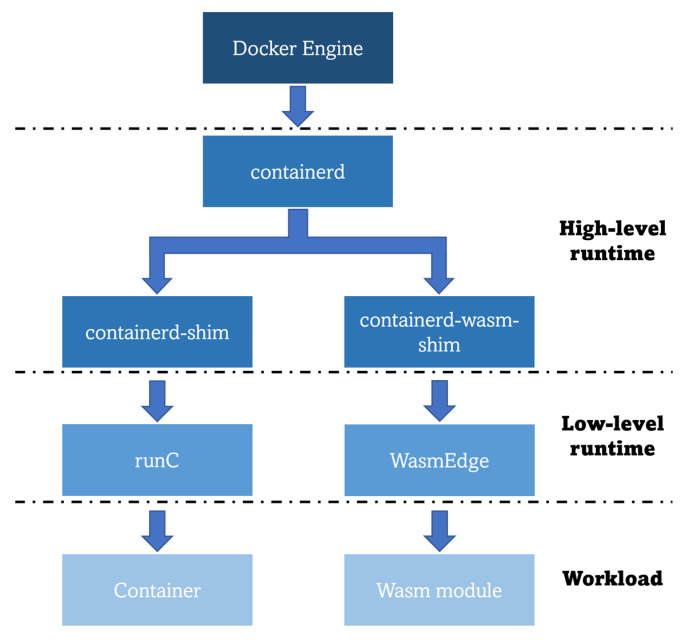
Docker Engine architecture for running Wasm workloads [110].

The hype about this technology is such that Docker cofounder Solomon Hykes stated in 2019, that if Wasm along with WASI had existed in 2008, they would not have needed to create Docker [108]. However, this does not mean that these two types of workloads are incompatible or that one technology is expected to replace the other in the future; both can be combined, and the choice of the working technology should be made depending on the final business use case. For instance, containers are a better fit for strong filesystem control and intensive I/O operations, while Wasm is better for setting up simple web servers [109]. In addition, Docker has recently announced the compatibility of the Docker Engine with Wasm through the usage of *WasmEdge* as its Wasm runtime for running such specific workloads. However, to do so, the developers must ensure that the Wasm modules are packaged as OCI-compliant images [110], so that the high-level runtime of the Docker Engine (*containerd*) can properly cope with the workload through the specifically created *containerd-wasm-shim*. Native compatibility is completed as this *shim* interacts with the *WasmEdge* Wasm runtime to run a Wasm application in the same way as it would interact with the low-level container runtime *runC* to run a container (as illustrated in Figure 7).

**Figure 8:**
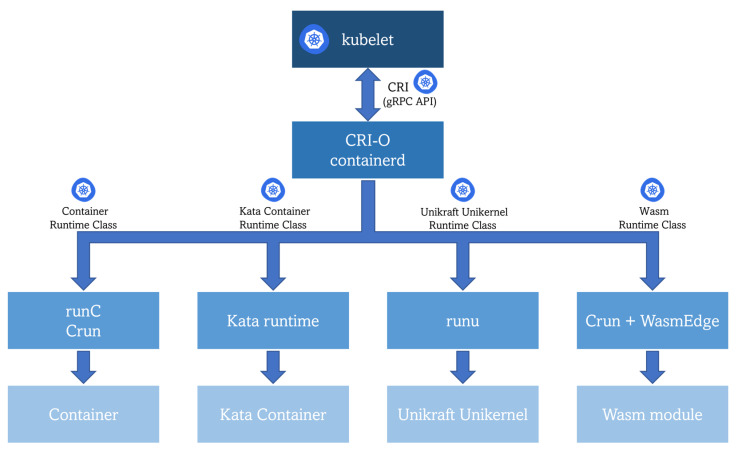
Running different types of workloads in K8s.

The coexistence of different types of workloads puts into the scene the requirement of a common orchestration mechanism. In Section 6, multiple technologies have been reviewed to prove that K8s is being increasingly adapted and successfully used as a container orchestration framework for the edge. The last part of this review article focuses on analyzing whether this powerful framework (K8s) could also be used to orchestrate other types of workloads on the edge. Observing the general architecture of Kubernetes, the *kubelet* component interacts with a high-level container runtime (also referred to as the CRI runtime or CRI implementation in K8s) that implements the Kubernetes Container Runtime Interface (CRI) acting as a gRPC API client for launching pods and their containers. Looking deeper into K8s functioning, a resource named *Runtime Class* is the key underlying component. This class is “a feature for selecting the container runtime configuration” [111], which can be leveraged for mapping different workload types with their corresponding low-level runtime (same architectural spot as the low-level runtime in Figure 1). Therefore, the installed high-level container runtime in the cluster, for instance, *CRI-O* or *containerd* (see Section 5), will be able to send a request to the proper low-level runtime in order to actually run the requested workload, as shown in Figure 8. The only requirement is packaging the application into an OCI-compliant image previously stored in an OCI-compliant image registry (same pre-condition as for the Docker engine).

Paying particular attention to Wasm module workloads in the K8s environment, *Krustlet* is an experimental project based on the rewrite of the *kubelet* component using the Rust language to run natively Wasm-based workloads. *Krustlet* relies on the use of K8s tolerations for scheduling such workloads to be deployed on K8s nodes, where the *Wasmtime* Wasm runtime had been previously installed [112]. As a final note, the relationship between K8s and Wasm modules is not limited to the deployment of Wasm applications instead of containers. Innovative studies like the one presented in [113] propose to reduce the K8s control plane overhead, one of the principal reasons for K8s’s heavy size, through the deployment of Wasm-based K8s operators (modules that extend K8s API features) on demand. In that work, the replacement of traditional container-based controllers with Wasm-based ones showed a reduced memory consumption of about 64%.

## 8. Conclusions

The cloud computing paradigm has proven a great success in the last few years, while, nowadays, edge computing challenges to replicate the advantageous traits posed by cloud (such as virtualization of workloads), moving them closer to the local action. In this regard, several initiatives have been analyzed, focusing on those related to the container virtualization mechanism. Among the most relevant findings, novel runtimes exist that evolve Docker Engine (current de facto standard), such as *crun* and *CRI-O*, as well as specific operating systems such as EVE-OS, BalenaOS or Pantacor.

The most relevant challenge lies in the orchestration of such workloads considering the heterogeneity of computing equipment present in edge scenarios. A strong research line is the creation of lightweight versions of Kubernetes, which is a very popular tool in the cloud. Solutions to the like of *K3s* or *MicroK8s* are the current blueprints for this approach that can be used in combination and need a centralized Kubernetes deployment resulting in multi-clusters. Nonetheless, another approach is also gaining popularity which advocates for adapting the K8s architecture to the edge requirements instead of just reducing K8s components’ size and footprint. The most promising here is *KubeEdge*, which already presents several successful large-scale trials and has been officially accepted under the umbrella of the CNCF as an Incubating project.

Nonetheless, containers do present certain disadvantages that are even worsened in edge environments, such as large image size, non-robust security, and low isolation in the case of multi-tenant applications, among others. Because of this, novel ways of virtualizing workloads have emerged that are of special relevance for achieving edge-native principles. The most important trend for the future of cloud-native workload execution at the edge is the use of WebAssembly modules. Wasm’s benefits perfectly suit edge computing needs, and several initiatives and open projects have been reviewed in this contribution concluding that this approach is envisioned as the frontrunner for the next generation of edge-cloud continuum scenarios, becoming a strong alternative to containers rather than a full replacement of them. This emergence of novel workloads has highlighted the need for a common orchestration mechanism, so some initiatives have been conducted under the umbrella of Kubernetes.

## Figures and Tables

**Table 1:** Comparison of technologies for container orchestration at the edge.

Technology	K8s Based	Edge Service Mesh	Edge Node Autonomy	Edge Supported CPU Architectures	Production-Stage Deployments	Responsible and Status	Popularity
KubeEdge	Yes	Yes, completely decentralized with EdgeMesh	Yes	x86_64, ARMv8, ARMv7	Hong Kong–Zhuhai–Macao bridge China Mobile’s industrial internet big data center	CNCF Incubating	5.5k stars 1.5k forks
OpenYurt	Yes	Yes, using Raven	Yes	x86_64, ARMv8, ARMv7	Hema Fresh’s customer goods store AI project Video cloud migration in transportation	CNCF Sandbox	1.4k stars 298 forks
SuperEdge	Yes	Yes, through ServiceGroups	Yes	x86_64, ARMv8	Not documented	CNCF Sandbox	881 stars 190 forks
Open Horizon	Yes	No	No	x86_64, ARMv8, ARMv7, ppc64le	IBM Edge Application Manager	LF Edge Stage 2	87 stars 62 forks
Baetyl	Yes	No	Temporary	x86_64, i386, ARMv8, ARMv7l	Not documented	LF Edge Stage 1	1.8k stars 318 forks
Project Flotta	Yes	No	No	x86_64, ARMv8	Not documented	Red Hat	18 stars 21 forks
Eclipse ioFog	No	Yes	Yes	x86_64, ARMv8, ARMv7	Not documented	Eclipse Foundation	270 stars 34 forks

**Table 2:** Comparison of Wasm runtimes.

Technology	Language Written	WASI	Supported Languages for Workload Embedding	Supported CPU Architectures	Production-Stage Runtime Deployments	Responsible	Popularity
Wasmer	Rust	Yes	C, C++, C#, Crystal, D, Dart, Go, Java, JavaScript, PHP, Postgres, Python, R, Ruby, Rust, OCaml	x86_64, ARMv8	crun, Fluence Labs	Wasmer Inc.	14.1k stars 607 forks
Wasmtime	Rust	Yes	Bash, C, C++, Go, .NET, Python, Ruby, Rust	x86_64, ARMv8	crun, Shopify, Fastly, DFINITY, InfinityOn, Fermyon, Mebark, SingleStore, Microsoft, wasmCloud	Bytecode Alliance	11.1k stars 882 forks
Wasm3	C	Yes	Arduino, C, C++, Go, JavaScript, Perl, Python, .NET, Nim, Swift, Zig	x86_64, x86_32, ARMv8, ARMv7, RISC-V, XTENSA, MIPS, ARC	Shareup, wasmCloud	CNCF Sandbox	5.7k stars 367 forks
WasmEdge	C++	Yes: Sockets, Crypto, Machine learning (wasi-nn), proxy-wasm	C, Go, Node.js, Python, Rust	x86_64, ARMv8	crun, Docker Engine, Suborbital scheduler and engine	CNCF Sandbox	4.9k stars 446 forks
WAMR	C	Yes	C, C++, Go, Python	x86_64, x86_32, ARMv8, ARMv7, RISC-V, XTENSA, MIPS, ARC	Not documented	Bytecode Alliance	3.3k stars 429 forks
wazero	Go	Yes	Go	x86_64, ARMv8, RISC-V64 interpreter	Not documented	Tetrate Labs	2.4k stars 129 forks
WAVM	C/C++	Yes	C, C++	x86_64	Not documented	CNCF Sandbox	2.3k stars 214 forks
wasmi	Rust	Yes	Rust	Not documented	Substrate blockchain	Parity Technologies	1k stars 198 forks

## Data Availability

No data were created or analyzed in this study. Data sharing is not applicable to this article.
